# Characterization and Antimicrobial Susceptibility of *Lactococcus lactis* Isolated from Endodontic Infections in Ouagadougou, Burkina Faso

**DOI:** 10.3390/dj6040069

**Published:** 2018-12-10

**Authors:** Wendpoulomdé Aimé Désiré Kaboré, René Dembélé, Touwendsida Serge Bagré, Ali Konaté, Sylvie Boisramé, Valérie Chevalier, Tarcissus Konsem, Alfred S. Traoré, Nicolas Barro

**Affiliations:** 1 Laboratory of Molecular Biology, Epidemiology and Surveillance of Bacteria and Viruses Transmitted by Food (LaBESTA)/Center for Research in Biological, Food and Nutritional Sciences (CRSBAN)/Graduate School of Science and Technology (EDST), University of Ouaga I, Professor Joseph KI-ZERBO, Ouagadougou 03 BP 7021, Burkina Faso; simavedemb@gmail.com (R.D.); sergebagre@gmail.com (T.S.B.); zangaali@gmail.com (A.K.); astraore@gmail.com (A.S.T.); barronicolas@yahoo.fr (N.B.); 2 Training and Research Unit in Health Sciences (UFR/SDS), University of Ouaga I, Professor Joseph KI-ZERBO, Ouagadougou 03 BP 7021, Burkina Faso; tarcissus@hotmail.com; 3 University Laboratory of Biodiversity and Microbial Ecology, EA 3882/University of Western Brittany, 22 av C. Desmoulins 29238 Brest CEDEX, France; sylvieboisrame@hotmail.com; 4 Department of Odontology Conservative and Endodontics, University of Western Brittany, 22 av C. Desmoulins 29238 Brest CEDEX, France; valerie.chevalierherisset@gmail.com

**Keywords:** *Lactococcus lactis*, endodontic infections, antimicrobial susceptibility

## Abstract

Background: This study aimed to characterize and test the antimicrobial susceptibility of *Lactococcus lactis* isolated in endodontic infections in Burkina Faso. Material and methods: This was a prospective study conducted at the Municipal Oral Health Center of Ouagadougou, Burkina Faso, from June to October 2014. Clinical data were collected using a questionnaire form. The method of streaking on selective medium was used to isolate bacteria. Identification was made using the API 20 Strep gallery. Antibiotic susceptibility was performed by the diffusion method on solid medium. Results: One hundred and twenty-five (125) patients were received with a significant proportion from the age group of 19 to 40 years (55.2%). Apical periodontitis accounted for 50.4% and cellulitis for 49.6% of cases. *Lactococcus lactis* ssp. *lactis* was identified in five exudate samples. Isolates were 100% resistant to cefixime and metronidazole, 80% to ceftriaxone, cefuroxime, cefotaxime, chloramphenicol and 60% to penicillin G, amoxicillin, amoxicillin clavulanic acid. A multidrug resistance of more than three families of antibiotics was noticed. No strains produced extended spectrum ß-lactamases. Conclusion: *Lactococcus lactis* is part of endodontic biofilm. The reported strong antibiotic resistance involving endodontic therapy will focus on the effect of the disinfectant solution and the mechanical action of the canal instruments.

## 1. Introduction

Apical periodontitis is mainly caused by a bacterial colonization of the canal space [1]. A direct complication of this apical periodontitis is apical abscess. If the primary source of the infection is not eliminated, the process of inflammation can progress and may result in severe (local as well as regional or systemic) complications as circumscribed cellulitis or cervico-facial cellulitis, etc. The main symptoms include pain, swelling and erythema [2]. To preserve a tooth the pulp of which has been exposed to bacteria, endodontic treatment must be performed. Endodontic reprocessing becomes necessary when there is a failure of the initial treatment, of which a principal manifestation is the appearance of secondary infections. The persistence of microorganisms or re-infections is the main cause of failure in endodontic treatment [3]. The bacterial colonization of the canal space has been shown to be the main etiological factor of endodontic infections. Studies have shown that bacterial biofilm is variable. Bacteria may therefore modify the severity and prognosis of endodontic infections [4,5]. Many studies have been carried out in the last ten years to understand the microbiota of the human oral cavity and endodontic microbiome [6,7]. The importance of precisely characterizing the endodontic microbiome no longer requires demonstration. Knowledge of the endodontic bacteria is necessary for a better understanding of the bacterial taxons involved in the inflammation process [8]. Lactic acid bacteria have been used for centuries to ferment foods and thus better preserve them [9]. Deemed harmless to humans, their use is widespread in the food industry. However, rare cases of infections of greater or lesser severity have been reported in humans [10], and although few in number, some studies have reported *Lactococcus lactis* in endodontic infections [11,12,13]. Koyuncu et al. (2005) [14] reported a deep neck infection due to *Lactococcus lactis* with the consumption of unpasteurized milk, occurring in a patient with a buccal mucosa tumor. Mussano et al. (2018) [1] showed a significant presence of *Lactococcus lactis* in periapical granulomas. *Lactococcus lactis* is an optional Gram-positive anaerobic coccus, sometimes isolated from human cutaneous surfaces (gut, mouth, vagina and skin surface) [15,16]. In Burkina Faso, oral pathologies are worrying. However, few works have been carried out to better understand endodontic microbiology. The objective of this study was to identify and determine antibiotic susceptibility of *Lactococcus lactis* in endodontic infections in Ouagadougou, Burkina Faso.

## 2. Materials and Methods

### 2.1. Study Design, Period and Settings 

**Figure 1:**
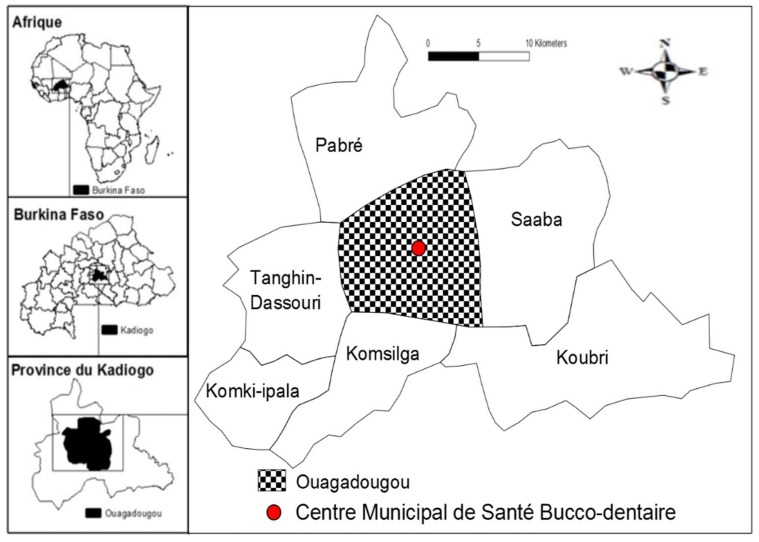
Map of Kadiogo Province with the study sites indicated.

This prospective study was performed from June to October of 2014 in Ouagadougou, Burkina Faso. Specimens were obtained at the Municipal Oral Health Center, Ouagadougou (MOHC) (Figure 1). Microbiological analyses were carried out at the Molecular Biology Laboratory for epidemiology and monitoring of food-borne bacteria and viruses at the Ouaga I Professor Joseph KI-ZERBO University.

### 2.2. Diagnostic Criteria for Endodontic Infections

Three key clinical criteria were used to diagnose apical periodontitis: The presence of an endodontic bacterial entryway; a negative response to pulp vitality tests; and a positive percussion pain when apical periodontitis was acute. Swelling in the front of the dental apex or an apical image were considered. The discoloration of the tooth was also noted. A diagnosis of cellulitis was based on exobuccal and endobuccal examinations. The exobuccal examination looked for maxillary and/or cervicofacial tumefaction, cutaneous inflammation, trismus and/or under angulomaxillary adenopathy. The endobuccal examination looked for an infectious portal of endodontic origin.

### 2.3. Inclusion and Non-Inclusion Criteria

Patients for whom an apical periodontitis or cellulitis of endodontic origin have been established were considered for sampling. Whether the tooth is permanent or temporary was not an exclusion criterion. Any tooth for which the root canal had already been filled, the presence of a periodontal pocket of 5 mm or more and an endobuccal or extraoral fistula were criteria for exclusion. Teeth where the pulp chamber was exposed to the oral cavity were also excluded from sampling. No medical history (patients with HIV, diabetes, cancer or patients on corticosteroids) was an exclusion criterion. In addition, patients who started antibiotic therapy only on the day of collection were included in the study. Patients were all examined by a dental surgeon.

## 3. Experimental Procedures

### 3.1. Patient Data Collection 

The civil data (age, gender, etc.) and medical history were collected using a form. The retention index of Björby and Löe [17] was used to assess the oral hygiene level (Table 1). Once the causative tooth had been identified, a retro-alveolar radiography was performed. Then, a periodontal sounding measured the depth of the pockets. Patients were classified into 3 occupational categories: (i) low income category (farmers, students, pupils, and housewives), (ii) high income category (business people and private sector employees), and (iii) patients with a moderate income (public sector employees, informal sector workers, retirees).

### 3.2. Sample Collection 

Sampling was carried out according to the method of Rôcas and Siqueira [18]. First, each patient rinsed their mouth for 30 s with a 0.12% chlorhexidine solution. Bacterial plaque was removed by disinfecting and cleaning the dental surfaces with 2% chlorhexidine tamped compresses. Then a rubber dam was set up and the endodontic access cavity is carried out using an endo access kit (Dentsply, York, PA, USA) in order to obtain exudates via root canals. Sterile paper tips that fit the root canal were used for sampling. The selected paper point (generally number 15) was introduced at the estimated working length to remove exudate. Sampling for abscessed apical periodontitis and cellulitis was performed by the aspiration of 2 mL purulent exudate with a mounted sterile syringe, prior to the inflated mucosa being sanitized with 2% chlorhexidine. The exudate samples were immediately transferred to a sterile tube containing an anaerobic broth, the resazurine thioglycolate (Liofilchem, Roseto degly Abruzzi, Italy). In dry pulp necrosis, 1 mL of resazurine thioglycolate was injected into the canal space and mixed with a number 15 adjusted file. Sampling was carried out using a sterile paper point that was left in the root canal for about 30 s. For a multi-rooted tooth, sampling was performed in the canal in contact with the apical infection. When several teeth were involved in the same patient, exudate samples were obtained from each tooth, and this was accounted for in the labeling process. Tubes were refrigerated in a cooler at 4 °C and transported to the laboratory for microbiological analysis within two hours.

### 3.3. Isolation and Identification of Lactococcus lactis 

From anaerobic transport broth, thioglycolate with resazurin (Liofilchem, Marcy-l’Étoile, Italy), a 0.5 Mc Farland aliquot (10 μL) was inoculated on M17 agar (Biokar Diagnostics) [19]. Petri dishes were incubated at 37 °C for 48–72 h in a jar containing Genbox (Liofilchem, Marcy-l’Étoile, Italy) to create partial anaerobiosis. Probable *Lactococcus* colonies (small colonies of 0.5 to 1 μm, white, round or lenticular in pairs or chains, catalase negative, positive hydrogen sulphide) were subcultured on Mueller-Hinton medium (Liofilchem, Marcy-l’Étoile, Italy) for confirmation of biochemical characterization using API 20 STREP gallery (bioMérieux, Marcy-l’Étoile, France). A reading was made according to the recommendations of the manufacturer then interpreted with Apiweb software version V7.0.

### 3.4. Antimicrobial Susceptibility Testing

An antimicrobial susceptibility test was carried out using the agar disc diffusion method as previously described by Bauer et al. (1966) [20]. Antibiotic disks were deposited on plates and then the plates were incubated anaerobically at 37 °C for 18–24 h. Twenty one antibiotics from eight different families were tested: Beta-lactams (oxacillin 5 μg, amoxicillin 30 μg, amoxicillin-clavulanic acid 20 + 10 μg, cefotaxime 30 μg, cefuroxime 30 μg, cefixime 5 μg, ceftriaxone 30 μg, piperacillin 100 μg, piperacillin-tazobactam 100 + 10 μg, penicillin G 10 IU); Macrolides (erythromycin 15 μg, spiramycin 100 μg); Sulfamides (trimethoprime-sulfamethoxazole 1.25/23.75 μg); Phenicols (chloramphenicol 30 μg); Aminosides (gentamicin 10 μg, tobramycine 10 μg, netilmicin 30 μg); Nitro-imidazols (metronidazole 5 μg); Lincosamides (lincomycin 15 μg, clindamycin (10 μg) and Quinolones (ciprofloxacin 5 μg) (Liofilchem, Italy). The areas of the inhibition diameters were recorded and classified as either “resistant (R)”, “intermediate (I)”, or “sensitive (S)” according to the French Microbiology Society Antibiogram Committee’s recommendations (FMSAC/EUCAST, 2017) [21]. The intermediate (I) susceptibility of the pathovars was considered as resistant (R).

### 3.5. Phenotypic Detection of Extended Spectrum ß-Lactamase (ESBL)

Strains that were β-lactam resistant were subjected to investigation of the extended spectrum ß-lactamase activity according to the recommendations of FMSAC/EUCAST (2017) [21]. The double synergy test was used for ESBL-producing strain detection. The discs of ceftriaxone and cefotaxime were placed around an amoxicillin-clavulanic acid disc on the bacterial plate. The distance between discs, center to center, was about 2 to 3 cm.

### 3.6. Conservation of Isolated Strains

After identification, *Lactococcus lactis* strains were maintained in thioglycolate cryotubes with resazurin (Liofilchem, Italy) and 60% glycerol. Cryotubes thus prepared were stored in the freezer at −20 °C.

### 3.7. Statistical Analyses

Statistical analyses were carried out using Sphinx Plus^2^ 5 software (Park Altai’s, 74650 Chavannes, France). The χ^2^ (Chi-squared) test was used to compare two qualitative variables. Differences were considered to be significant when *p* < 0.05.

### 3.8. Ethical Considerations

The research protocol was approved by the national ethics committee of Burkina Faso prior to the study. Approval Deliberation N° 2009-30 was issued on 17 July 2009. Data were collected with informed patient consent.

## 4. Results

### 4.1. Characteristics of Patients

One hundred twenty-five patients were examined, including 62 males (49.6%) and 63 females (50.4%) (*p* = 0.9287). There was a fairly high number of cases in patients aged between 19 and 40 years (55.2%) (*p* = 0.0001). Sixty-two had dental cellulitis (49.6%) and 63 had apical periodontitis (50.4%). Cellulite accounted for 41.6% and apical periodontitis 32.8% of disease at the acute stage. In the chronic stage, there was 8% cellulitis and 17.6% apical periodontitis. The differences were very significant between the two infectious stages (*p* = 0.0001). Low-income patients (farmers, students, and housewives) (47.2%) were the most affected (*p* = 0.0001). Low-income earners (public sector employees, informal sector workers, retirees and others) accounted for 27.2% and high-income earners (private sector employees and traders) for 25.6% of patients. Poor oral hygiene was present in 84.8% of patients, who were assigned a score of 3 (*p* = 0.0001). Patients consumed mostly fish products (smoked fish) (39.2%) and meat products (38.4%).

### 4.2. Prevalence of Isolated Bacteria

Five exudate samples (4%) were positive for *Lactococcus lactis*. *Lactococcus lactis* ssp. *lactis* was the only subspecies that was isolated from these five samples, including two cases of facial cellulitis and three cases of apical periodontitis. Other bacteria were identified in two samples. These were *Streptococcus mitis*, *Aerococcus viridans*, *Aerococcus urinae* and *Gemella haemolysans* in the first sample and *Streptococcus uberis* in the second sample.

### 4.3. Antibiotic Susceptibility Profile

The strains of *Lactococcus lactis* ssp. *lactis* showed overall strong resistance. They were resistant (100%) to cefixime and metronidazole, 80% to ceftriaxone, cefuroxime, cefotaxime, chloramphenicol and 60% to penicillin G, amoxicillin, amoxicillin-clavulanic acid. The sensitivity to all antibiotic isolates tested was low. This was 60% for gentamicin, clindamycin, piperacillin-tazobactam, tobramycin lincomycin and piperacillin (Table 2). Multidrug resistance to more than three families of antibiotics was noticed.

## 5. Discussion

During the last decade, many studies have focused on lactic acid bacteria, because of their potential use in the food and health sectors [22,23]. In addition, the field of investigation has largely focused on potential therapeutic applications [19,24,25]. Although *Lactococcus lactis* is primarily known to be non-pathogenic, the pathogenicity of this agent should be kept in mind. Indeed, recent cases of *Lactococcus lactis* infections have been reported [10,16,26]. One hundred and twenty-five samples were collected from patients, of whom 62 were male (49.6%) (*p* = 0.9287). The age group between 19 and 40 years (55.2%) (*p* = 0.0001) is the largest. Previous studies reported the same trends [27,28,29]. Our study shows that low-income populations are most strongly affected by endodontic infections. Vulnerable groups are most frequently exposed to dental disease [30,31]. Our study used standard phenotypic methods to identify isolates. It was shown that *Lactococcus lactis* can be found in endodontic infections. *Lactococcus* was for a long time confused with group D fecal streptococci and included in the Streptococcus group [10]. In our study, isolated strains were multi-resistant. Antimicrobial sensitivity was not good for beta-lactamines and particularly for 3rd generation cephalosporins. It was 60% for lincosamides tested. This sensitivity was found to be 60% for tobramycin and 40% for netilmicin. A study reported cases of *Lactococcus lactis* spp. *lactis* infections in children that were successfully treated with vancomycin. The authors showed that *Lactococcus lactis* spp. *lactis* was sensitive to penicillin and clindamycin [10]. A recent study reports a case of *Lactococcus lactis* spp. *lactis* endocarditis. The isolate was sensitive to ampicillin, ceftriaxone, clindamycin, chloramphenicol, erythromycin and oxacillin [32]. The authors report a dental history of the patient who had dental implants, with the last visit to the dentist was six months earlier. In addition he lived in a village where he cared for chickens, rabbits and lambs. A focal dental infection could therefore not be excluded. Although cases of *Lactococcus lactis* spp. *lactis* infections are rare, this bacterium has been linked to serious, fatal infections [33,34,35,36]. These cases of infection most often affect non-immunocompetent people [32]. Some authors report natural resistance to aminoglycosides [37,38]. However, the origin and mechanism of the contamination are not yet well known. Contamination in humans may occur from contact with unpasteurized dairy products [10,14] or through contact with animals (sheep, chickens and rabbits) by hand-to-mouth contamination [32]. *Lactococcus lactis* spp. *lactis* would infect livestock and ranchers, however, the route of infection is still unclear. Some studies in Burkina Faso show a significant consumption of meat and milk, which could explain the contamination. Additionally, these studies report the uncontrolled use of antibiotics in the animal industry [39,40]. Industries and hospitals, through their discharge of effluent and solid waste into the environment without adequate treatment, are a source of pollution of ecosystems [39,41]. The pollution of surface waters by heavy metals, pesticides, fertilizers, hydrocarbons and pathogenic organisms is a real environmental and health problem. All this contributes to amplifying the phenomena of antibiotic resistance and the emergence of multidrug-resistant bacteria [42]. This situation could also explain the multidrug resistance of the strains identified in our study. The epidemiology and clinical picture of this emerging pathogen remains largely unknown. A search of the literature found very few studies reporting *Lactococcus lactis* in oral infections. This is certainly due to confusion regarding its identification and its being confused with other bacterial genera. Understanding microbiology, particularly biofilm biology, is an essential element for the creation of targeted, effective and efficient therapeutic modalities [43]. The antibiotic susceptibility study of the five isolated strains showed resistance to most of the β-lactams and macrolides that are common in odontology. Teeth could end up being the seat of dissemination to noble organs [44]. Focal infections of dental origin mean that a dental infection can cause distant lesions. However, it remains difficult to prove absolutely the oral origin of the bacteria responsible for a focal infection [45].

## 6. Conclusions

Pulpal and periradicular infections are known to be mediated by biofilm, which provides resistance to microbial flora. The structure of the biofilm as well as the dynamics of the interactions between bacteria provide this resistance. *Lactococcus lactis* spp. *lactis* is now known to be part of this endodontic biofilm. Endocanalar bacteria can be eradicated through the mechanical action of canal instruments and the effects of a disinfectant solution. Several studies have reported that tooth decay was the leading cause of consultation at dental practices in Burkina Faso. The effective prevention of carious disease requires the involvement of primary care providers dispensing essential skills and tools, information, and education activities promoting behavioral change in oral health care. The real involvement of public authorities is necessary, especially in the implementation of health insurance for all.

## Figures and Tables

**Table 1:** Oral hygiene index.

0	1	2	3
Absence of tartar, tooth decay or fillings	Caria, scale or shutter close to the gum	Caria, tartar, or filling in contact with the marginal gingiva, a degree of subgingival calculus	Caria, tartar, or filling in the marginal gingiva, abundant subgingival calculus

Legend: 0 = Score of zero, **1** = Score of one, **2** = Score of two, **3** = Score of three.

**Table 2:** Antimicrobial susceptibility of *Lactococcus lactis* ssp. *lactis* isolates.

Antibiotics	Susceptibility of Isolates N (%)	
	Resistant (R + I)	Sensitive
Amoxicillin-clavulanic acid	3 (60)	2 (40)
Ceftriaxone	4 (80)	1 (20)
Cefixime	5 (100)	0 (0)
Cefuroxime	4 (80)	1 (20)
Cefotaxime	4 (80)	1 (20)
Gentamycin	2 (40)	3 (60)
Clindamycin	2 (40)	3 (60)
Metronidazole	5 (100)	0 (0)
Piperacillin-tazobactam	2 (40)	3 (60)
Oxacillin	4 (80)	1 (20)
Spiramycin	3 (60)	2 (40)
Lincomycin	2 (40)	3 (60)
Piperacillin	2 (40)	3 (60)
Tobramycin	2 (40)	3 (60)
Netilmicin	3 (60)	2 (40)
Erythromycin	3 (60)	2 (40)
Trimethoprim-sulfamethoxazole	3 (60)	2(40)
Chloramphenicol	4 (80)	1 (20)
Ciprofloxacin	3 (60)	2 (40)
Penicillin G	3 (60)	2 (40)
Amoxicillin	3 (60)	2 (40)
